# Dynamic regulation of mitochondrial-endoplasmic reticulum crosstalk during stem cell homeostasis and aging

**DOI:** 10.1038/s41419-021-03912-4

**Published:** 2021-08-16

**Authors:** Weiping Lin, Shuxun Chen, Yan Wang, Ming Wang, Wayne Yuk-Wai Lee, Xiaohua Jiang, Gang Li

**Affiliations:** 1 Centre for Regenerative Medicine and Health, Hong Kong Institute of Science & Innovation, Chinese Academy of Sciences, Hong Kong SAR, China; 2 Stem Cells and Regenerative Medicine Laboratory, Department of Orthopaedics and Traumatology, Li Ka Shing Institute of Health Sciences, Prince of Wales Hospital, The Chinese University of Hong Kong, Hong Kong SAR, China; 3 Department of Biomedical Engineering, City University of Hong Kong, Hong Kong SAR, China; 4 SH Ho Scoliosis Research Laboratory, Joint Scoliosis Research Center of the Chinese University of Hong Kong and Nanjing University, Department of Orthopaedics and Traumatology, The Chinese University of Hong Kong, Hong Kong SAR, China; 5 Shenzhen Research Institute, The Chinese University of Hong Kong, Shenzhen, China; 6 Faculty of Medicine, MOE Key Laboratory for Regenerative Medicine, School of Biomedical Sciences, The Chinese University of Hong Kong, Hong Kong SAR, China

**Keywords:** Energy metabolism, Stem-cell research

## Abstract

Cellular therapy exerts profound therapeutic potential for curing a broad spectrum of diseases. Adult stem cells reside within a specified dynamic niche in vivo, which is essential for continuous tissue homeostatic maintenance through balancing self-renewal with lineage selection. Meanwhile, adult stem cells may be multipotent or unipotent, and are present in both quiescent and actively dividing states in vivo of the mammalians, which may switch to each other state in response to biophysical cues through mitochondria-mediated mechanisms, such as alterations in mitochondrial respiration and metabolism. In general, stem cells facilitate tissue repair after tissue-specific homing through various mechanisms, including immunomodulation of local microenvironment, differentiation into functional cells, cell “empowerment” via paracrine secretion, immunoregulation, and intercellular mitochondrial transfer. Interestingly, cell-source-specific features have been reported between different tissue-derived adult stem cells with distinct functional properties due to the different microenvironments in vivo, as well as differential functional properties in different tissue-derived stem cell-derived extracellular vehicles, mitochondrial metabolism, and mitochondrial transfer capacity. Here, we summarized the current understanding on roles of mitochondrial dynamics during stem cell homeostasis and aging, and lineage-specific differentiation. Also, we proposed potential unique mitochondrial molecular signature features between different source-derived stem cells and potential associations between stem cell aging and mitochondria–endoplasmic reticulum (ER) communication, as well as potential novel strategies for anti-aging intervention and healthy aging.

## Facts

- Stem cell niche is essential for cell-fate decisions via regulation of stem cell homeostasis and mitochondrial dynamics of fusion and fission through balancing self-renewal and lineage-specific differentiation, as well as stem cell quiescence and activation.
- Circulating cell-free mitochondria exist within the peripheral blood, which may be involved in various pathophysiological processes.
- Mitochondria are highly dynamic organelles that change their morphology in response to cellular signals and differentiation states.
- Mitochondria–endoplasmic reticulum crosstalk is implicated in aging progression.
- Asymmetrically sort and distribution of aged and young mitochondria are critically involved in stemness regulation of stem cells.

## Open questions

- Is mitochondrial functional decline involved in aging progression of stem cells through age-dependent subcellular localization and redistribution of the mitochondria, thereby causing loss of stem cell properties?
- Are mitochondrial connections and transfer implicated in cell-based therapies via sharing and receiving of energetic and young mitochondria of cells of damaged tissues from functional stem cells?
- Is the coupling of endoplasmic reticulum (ER) stress and cell differentiation associated with the interplay between mitochondrial–ER crosstalk?
- Is it possible to alleviate stem cell aging or achieve rejuvenation of aging stem cells through switching prolonged or excessive endoplasmic reticulum (ER) stress to adaptive ER stress via regulation of mitochondrial function and stem cell niche?

## Introduction

Stem cell-based therapies exert profound therapeutic potential for curing a broad spectrum of diseases [1, 2]. Adult stem cells reside in a specified dynamic niche that is essential for continuous tissue homeostatic maintenance through balancing self-renewal with lineage selection [3, 4]. Meanwhile, adult stem cells may be multipotent or unipotent, and are present in both quiescent and actively dividing states in vivo of the mammalians, which may switch to each other state in response to various intrinsic or extrinsic signals through mitochondria-mediated mechanisms, such as alterations in mitochondrial respiration and metabolism [4–9]. In addition, the co-incidence of endoplasmic reticulum (ER) and mitochondria, and their dynamic interconnections and crosstalk are involved in a series of cellular processes, including mitochondrial homeostasis in fusion and fission, autophagy, and inflammasome formation [10, 11].

Emerging novel techniques during recent years, such as single-cell transcriptomics for analysis of spatial and temporal turnover of certain cellular processes, may enable advancing our understanding of dynamic gene regulation in stem cell maintenance within stem cell niche, stem cell activation and mobilization, lineage specification, tissue-specific molecular phenotypes in adult stem cells, identification of major cell types and their localization, as well as cellular and spatial sources of key growth factors and cytokines [12–18].

### Diversity and heterogeneity of mitochondria

**Fig. 1:**
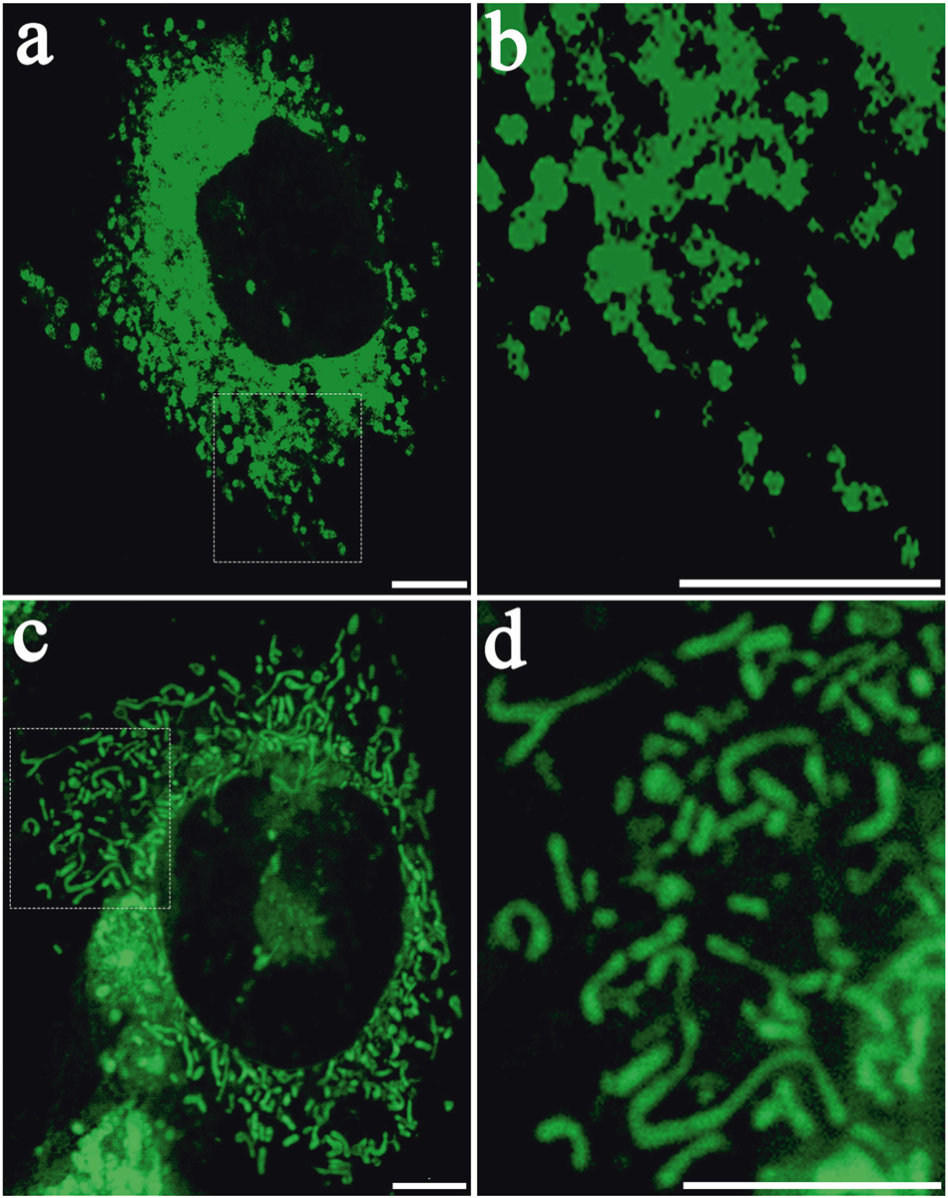
Representative images of mitochondrial morphology. Observation of mitochondria of murine peripheral blood-derived mesenchymal stem cells (mPB-MSCs) under confocal microscope through Mito-Tracker Green staining. **a**, **b** Representative images of mitochondria of undifferentiated mPB-MSCs. **c**, **d** Representative images of mitochondria during osteogenic differentiation. Scale bar: 10 µm.

Mitochondria are complex organelles existing in a network undergoing continuous morphological dynamic changes through fission and fusion, which is crucial for the maintenance of pluripotency and differentiation capacity of stem cells [19–24]. Mitochondria usually undergo continuous morphologic dynamic changes through fission and fusion events controlled by the large GTPases Drp1, Mfn1, Mfn2, and Opa1 (fusion), which are important for mitochondrial function, and their imbalance would cause cell dysfunction and various diseases [25–28]. Metabolic changes are essential for cell-lineage commitment during mesenchymal stem cell differentiation, accompanied with alterations in mitochondrial morphology and dynamics [21, 22, 29]. Distinct morphological characteristics of mesenchymal stem cells mitochondria occur during different lineage-specification state (Fig. 1).

**Fig. 2:**
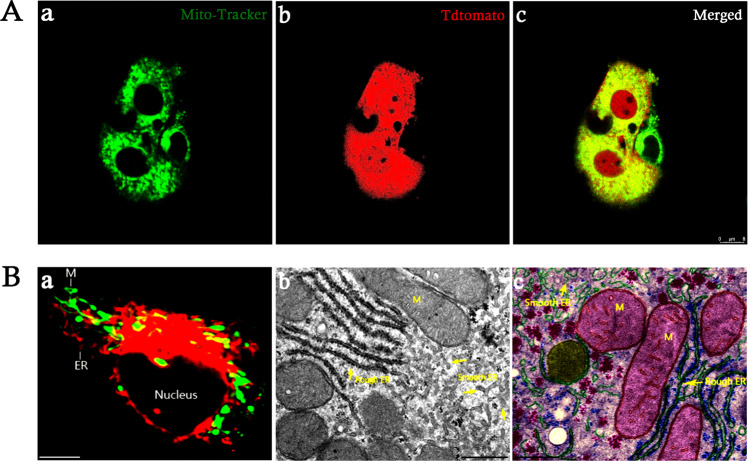
Endoplasmic reticulum-mitochondrial localization. **A** Representative live mitochondrial images of tdTomato-labeled joint progenitor cells co-cultured with mature chondrocytes through Mito-Tracker Green staining under confocal microscope. **a** Representative live-cell imaging of mitochondria through Mito-Tracker Green staining; **b** representative live-cell imaging of tdTomato fluorescence; **c** merged images. Scale bar: 8 µm. **B** Structural features of mitochondria (M) and endoplasmic reticulum (ER) within cells. **a** Representative immunocytochemical images of M (green) and ER (red); scale bar: 10 µm. **b** Representative electron micrographs of M and ER; scale bar: 500 nm. **c** Representative images of ultrastructure of M and ER. Scale bar: 500 nm (adapted from netterimages.com and chegg.com).

In general, stem cells facilitate tissue repair after tissue-specific homing through various mechanisms, such as immunomodulation of local microenvironment, differentiation into functional cells [30, 31], cell “empowerment” via paracrine secretion [32–34], immunomodulation [35, 36], and intercellular mitochondrial transfer [37–40]. Recent studies have demonstrated intercellular mitochondrial transfer within osteocyte-dendritic network [41]. Intriguingly, potential mitochondrial connections and communications were also observed between co-cultured mature chondrocytes and stem cells ex vivo (Fig. 2A). Meanwhile, cell-source-specific features have been reported between different tissue-derived adult stem cells with distinct functional properties [5, 42], as well as differential functional properties in different tissue-derived stem cell-derived extracellular vehicles [43, 44], mitochondrial metabolism [45], and mitochondrial transfer capacity [46]. Strikingly, recent studies have reported the presence of circulating cell-free mitochondria within the peripheral blood, suggesting the diversity of existing forms of mitochondria [47, 48]. Therefore, it is highly possible that distinct mitochondrial gene expression patterns may exist between different tissue-derived stem cells, owing to the heterogeneity of stem cells and diverse populations of mitochondrial DNA (mt-DNA) [48–55]. Simultaneously, visualization of replicating mt-DNA nucleoids has suggested the physical linkage between the ER and mitochondria (Fig. 2B) [56–58]. Therefore, manipulation of mt-DNA within cells may represent a powerful approach for the development of therapeutic interventions to treat mitochondrial diseases.

### Mitochondrial metabolic regulation on stem cell fates

It is generally believed that stem cells fuel tissue development and tissue repair, and these activities are controlled by the local stem cell microenvironment or niche. Highly heterogeneous populations of resident stem/progenitor cells have been demonstrated residing within adult organs and tissues [55, 59–62]. An appropriate balance between self-renewal and differentiation is essential for stem cell function during both development and tissue homeostasis throughout life [63]. At steady state, adult stem cells are quiescent cells within niche. Both cell-intrinsic and -extrinsic signaling networks, such as mitochondrial dynamic-associated signaling, have been reported to fine-tune the self-renewal and differentiation of stem cells, and are involved in tissue homeostasis and tissue repair [64, 65].

Notably, mitochondrial plasticity, such as mitochondrial metabolism and mitochondrial respiratory chain, is vital for cell-fate decisions and function of stem cells [66–69]. The metabolic switch of mitochondria is required for stem cell activation and cell cycle activity [70]. Meanwhile, accumulating evidence has suggested a causative association between mitochondrial dysfunction and major phenotypes associated with aging. The self-renewal of tissues and organs in aging organisms requires stem cells, which have the unusual ability to divide asymmetrically into one daughter cell that retains stem cell properties and another that differentiates into a particular tissue type. Further, mitochondria have been reported to distribute passively during mitosis upon their release from microtubules [71]. Importantly, subcellular localization and distribution of young and old mitochondria determine stemness properties in the progeny stem cells during asymmetric cell divisions. Subsequently, the daughter cells that retains a stem cell nature inherits young mitochondria, whereas older mitochondria are inherited by the more differentiated cells [72]. Accumulation of aged mitochondria would lead to cell aging and cellular functional decline [70, 73]. Mohrin et al. [74] further elucidated a regulatory branch of mitochondrial unfolded protein response (UPR^mt^) that is coupled to cellular energy metabolism and proliferation in stem cells. Mitochondrial protein-folding stress triggered a metabolic checkpoint regulating cell cycle, whereas deregulation of this pathway interfered with stem cell quiescence and compromised regenerative potential [74]. Therefore, mitochondrial function may represent an important determinant of the regenerative potential of stem cells.

Stem cells possess multi-differentiation potential into various cell types, making them medically relevant for the treatment of a variety of diseases and injuries. However, there remains a major hurdle of stem cell therapy into the clinics, namely the limited efficiency to create fully functional and specialized terminally differentiated cells. Mitochondrial dynamics is crucial for cell-fate determination of stem cells [75, 76]. Importantly, different cell states require specific metabolic demands to support specialized functions [77]. Thus, efficient mitochondrial oxidative metabolism and dynamics are required for efficient specific lineage commitment [21, 78–83].

**Fig. 3:**
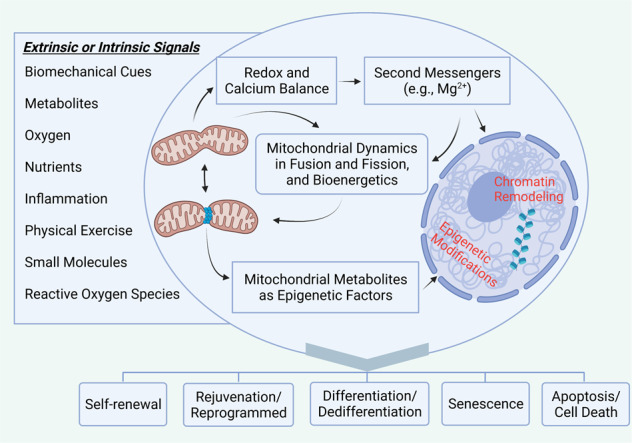
Mitochondrial regulation of stem cell homeostasis and aging. Regulation of stem cell homeostasis in response to environmental cues and epigenetic factors. Upon exposure to various extrinsic or intrinsic signals, mitochondria respond through modulation of morphological network and bioenergetics, the redox and calcium balance, and epigenetic modifications and chromatin remodeling within stem cells. Cell-fate decisions occur following mitochondria-based cellular response, mainly including self-renewal, rejuvenation/cell reprogrammed, differentiation/dedifferentiation, and senescence, cell death, or apoptosis [65, 134–142]. Created with BioRender.com.

Strikingly, recent studies report that chaperone-mediated autophagy and a related metabolite in embryonic stem cells, also known as the self-eating process, have emerged as promising novel therapeutics for regeneration of damaged tissues and organs [84]. Simultaneously, accumulating evidence has indicated tight associations between mitochondrial metabolism and stem cell differentiation [85, 86]. Studies have demonstrated that mouse embryonic stem cells sorted for low- and high-resting mitochondrial membrane potential (ΔΨ*mL* and ΔΨ*mH*) are indistinguishable in terms of morphology and expression levels of pluripotency markers, whereas differing markedly in metabolic rates, suggesting that a coupling between intrinsic metabolic parameters and stem cell fate may provide clues for novel enrichment strategies and therapeutic approaches of stem cell therapy [87, 88]. Furthermore, a recent study demonstrated lactate mobilization of intracellular Mg^2+^, indicating potential links between mitochondrial Mg^2+^ transportation with major metabolic feedback circuits and mitochondrial bioenergetics (Fig. 3) [89].

### Correlations between mitochondrial functions and aging

Adult stem cells are essential for tissue homeostasis and regeneration, yet are susceptible to senescence during aging [90–92], accompanied with aging microenvironments around adult stem cells [93, 94]. The main hallmarks of aging in mammalian organisms include genomic instability, telomere attrition, epigenetic alterations, loss of proteostasis, deregulated nutrient sensing, mitochondrial dysfunction, cellular senescence, stem cell exhaustion, and altered intercellular communication [95]. The degeneration or dysfunction of aging tissues and organs is attributed to the deterioration of adult stem cells [95–97], which may result from disordered mitochondrial dynamics and declined mitochondrial functions [98–100]. Mitochondrial activity and metabolism are important determinants for specification of stem cell fate [87, 101].

Studies have demonstrated the importance of oxidized form of cellular nicotinamide adenine dinucleotide on mitochondrial activity as a pivotal switch to modulate muscle adult stem cell senescence [102, 103]. SIRT3, a mammalian sirtuin that regulates the global acetylation landscape of mitochondrial proteins and reduces oxidative stress, is suppressed during aging. In addition, the upregulation of SIRT3 in aged hematopoietic stem cells (HSCs) improved the regenerative capacity of HSCs [104]. Also, maintenance of self-renewal of a purified Tie^2+^ HSC population relies on mitochondrial clearance [105]. Further studies have identified a regulatory branch of the UPR^mt^, which mediated through the interplay between SIRT7 and NRF1, which is coupled to cellular energy metabolism and proliferation. Deregulation of a UPR^mt^-mediated metabolic checkpoint as a reversible main factor for HSC aging [74]. Further, systemic chronic inflammation has been reported as an important feature of aging, which is critically implicated in the process of stem cell aging [106–108]. A recent study has uncovered mitochondrial stress-initiated aberrant activation of the NLRP3 inflammasome as a reversible driver of functional decline during HSC aging [109]. In the meanwhile, studies have documented the crucial roles of PTPMT1 (a PTEN-like mitochondrial phosphatase) in the metabolic regulation of self-renewal and differentiation of HSCs [110].

### Mitochondria–ER crosstalk and aging

**Fig. 4:**
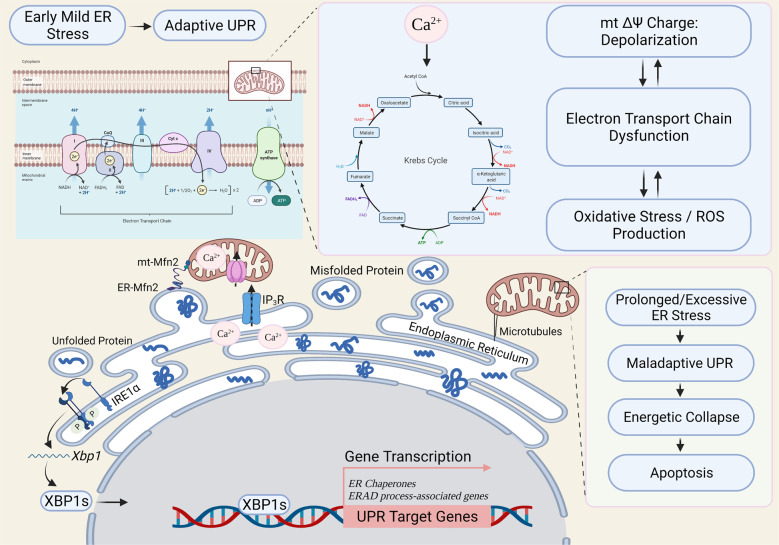
Proposed working model of communication between mitochondria and endoplasmic reticulum stress. Endoplasmic reticulum (ER) stress triggers an increase in mitochondrial metabolism, which mainly relies on organelle coupling and Ca^2+^ transfer. The onset of ER stress is accompanied with redistribution of reticular and mitochondrial networks towards the perinuclear region and a microtubule-dependent increase in connection. Physical interaction is mainly achieved by anchoring proteins, such as Mitofusin 2 (Mfn2), which allows buffering of intracellular Ca^2+^ from ER to mitochondria through its endoplasmic reticulum–mitochondria tethering activity, enhancing mitochondrial bioenergetics and ATP production consequently. The unfolded protein response (UPR) is a cellular self-defense adaptive mechanism to restore ER homeostasis. Crosstalk between the UPR pathways could facilitate a coordinated response to conditions of ER stress. During early mild ER stress, activated IRE1α then removes a 26-base intron from *Xbp1* mRNA to generate a potent transcription factor XBP1s (*Xbp1* spliced) that translocates into the nucleus and regulates a diverse array of genes, such as ER folding chaperones and ER-associated degradation (ERAD) process-associated genes. However, prolonged or excessive ER stress (e.g., induced by aging) would cause mitochondrial collapse and apoptotic cell death. ER: endoplasmic reticulum; UPR: unfolded protein response; ROS: reactive oxygen species; Mfn2: mitofusin 2; mt: mitochondrial; IRE1α: inositol-requiring kinase 1α (ER stress sensor); XBP1: X-box-binding protein 1; XBP1s: spliced form of XBP1; Krebs cycle: also known as TCA cycle (tricarboxylic acid cycle); IP_3_R: inositol trisphosphate receptor (Ca^2+^ channels); mt ΔΨ: mitochondrial membrane potential; ERAD: ER-associated degradation; ATP: adenosine 5´-triphosphate [74, 116,117,134, 142–145]. Created with BioRender.com.

Notably, surviving ER stress has been demonstrated coupling to altered chondrocyte differentiation and functioning, which facilitates survival and recovery through adaptive unfolded protein response (UPR) during pathophysiology of chondrodysplasia [111, 112]. Also, studies have suggested the intimate linkage between stress adaptation and aging process [113]. Stress responses and the aging process may share common features and mechanisms initially arising from studies in model organisms [114, 115], where various molecular pathways have been demonstrated to implicate in the progression of aging process, including insulin/insulin-like growth factor, sirtuins, targets of rapamycin (TORs), and AMP-activated kinase. Thus, intrinsic induction of stress defense programs and the resulting adaptation may become a potential strategy to increase life expectancy [114]. Usually, mild ER stress activates the adaptive UPR on the one hand. Adaptive UPR is conducive to stress alleviation in response to cellular stress, which has recently been reported to preserve self-renewal of hematopoietic and pre-leukemic HSCs via inositol-requiring enzyme 1α/X-box-binding protein 1 signaling [116]. On the other hand, however, beyond a certain degree of ER damage, namely prolonged UPR response, would trigger apoptotic pathways (Fig. 4) [117–120]. Studies have suggested that depletion of the proteins involved in the regulation of mitochondrial–ER crosstalk, such as mammalian TOR, would lead to increased apoptosis, autophagy, and cellular dysfunction [121, 122]. In contrast, artificially increasing ER–mitochondria contacts in cells would restore cell viability [89, 119, 123, 124]. Studies have further identified critical roles of ER–mitochondria contacts in the biogenesis of mitochondrial-derived compartments [125–127], which may play essential roles in cellular adaptation to environmental stress conditions [127].

Importantly, aging is one of the main causing factors of the increased prolonged ER stress, accompanied with mitochondrial dysfunction consequently [128]. Thus, attenuation of ER stress is a potential approach for the improvement and restoration of mitochondrial function in aging organisms. In addition, studies have suggested the correlation between spatial re-organization of mitochondria and increased ATP levels, oxygen consumption, reductive power, and increased mitochondrial Ca^2+^ uptake [129, 130]. However, uncoupling of the organelles or blocking Ca^2+^ transfer impaired the metabolic response, rendering cells more vulnerable to ER stress [129, 131]. Consequently, ER stress induces an early increase in mitochondrial metabolism that depends crucially upon organelle coupling and Ca^2+^ transfer, which, by enhancing cellular bioenergetics, establishes the metabolic basis for the adaptation to this response [129, 132]. As aging is one of the main factors causing increased ER stress and mitochondrial dysfunction, attenuation of ER stress is conducive to anti-aging [128]. Therefore, enhanced mitochondrial biogenesis has been reported associated with improved efficiency of the electron-transport chain, which may become a potential therapeutic anti-aging approach to block reactive oxygen species accumulation and promote cell survival through alleviation of ER stress [133].

## Conclusions

All together, mitochondrial plasticity plays central roles in regulation of activity and functions of stem cells. Intrinsic and extrinsic signaling networks are responsible for dynamic regulation in mitochondrial function and adaptation to intrinsic and extrinsic signals for ultimate cell fate decisions. Interplay and crosstalk among aging microenvironments, ER stress, and inter- and intracellular mitochondrial dynamics are implicated in the progression of stem cell aging and functionally declined tissues and organs. Further extensive investigations on mitochondria–ER communication-associated stem cell aging, and changes of chromatin states and mitochondrial dynamics within the regenerative niche will not only boost the development of novel pharmaceutical targets for the cure of age-related disorders through targeting mitochondria–ER associated signaling pathways, but also provide novel insights into mitochondria-mediated stem cell activation during tissue regeneration. Identification of specific mitochondrial molecular signatures between different source-derived stem cells may advance our understanding of stem cell biology and shed light on novel strategies for healthy longevity and improved therapeutic outcomes of cellular therapy.
